# Supplementary material for: Personalized mood prediction from patterns of behavior collected with smartphones
Source: NPJ Digit Med. 2024 Feb 28;7:49. doi: 10.1038/s41746-024-01035-6 (PMC10902386; doi:10.1038/s41746-024-01035-6)
Supplement: Supplementary file 2 — Reporting Summary [file 41746_2024_1035_MOESM2_ESM.pdf]

nature portfolio

Corresponding author(s): Brunilda Balliu and Jonathan Flint

Last updated by author(s): Aug 17, 2023

Reporting Summary

Nature Portfolio wishes to improve the reproducibility of the work that we publish. This form provides structure for consistency and transparency in reporting. For further information on Nature Portfolio policies, see our Editorial Policies and the Editorial Policy Checklist.

Statistics

For all statistical analyses, confirm that the following items are present in the figure legend, table legend, main text, or Methods section.

n/a	Confirmed	
☐	☒	The exact sample size (*n*) for each experimental group/condition, given as a discrete number and unit of measurement
☐	☒	A statement on whether measurements were taken from distinct samples or whether the same sample was measured repeatedly
☐	☒	The statistical test(s) used AND whether they are one- or two-sided<br>*Only common tests should be described solely by name; describe more complex techniques in the Methods section.*
☐	☒	A description of all covariates tested
☐	☒	A description of any assumptions or corrections, such as tests of normality and adjustment for multiple comparisons
☐	☒	A full description of the statistical parameters including central tendency (e.g. means) or other basic estimates (e.g. regression coefficient) AND variation (e.g. standard deviation) or associated estimates of uncertainty (e.g. confidence intervals)
☐	☒	For null hypothesis testing, the test statistic (e.g. *F*, *t*, *r*) with confidence intervals, effect sizes, degrees of freedom and *P* value noted<br>*Give P values as exact values whenever suitable.*
☒	☐	For Bayesian analysis, information on the choice of priors and Markov chain Monte Carlo settings
☒	☐	For hierarchical and complex designs, identification of the appropriate level for tests and full reporting of outcomes
☐	☒	Estimates of effect sizes (e.g. Cohen's *d*, Pearson's *r*), indicating how they were calculated

Our web collection on statistics for biologists contains articles on many of the points above.

Software and code

Policy information about availability of computer code

Data collection	Sensor data was collected using the AWARE framework
Data analysis	All statistical data analyses were performed with the R statistical software. The code that supports the findings of this study is available online at https://github.com/BrunildaBalliu/stand_mood_prediction.

For manuscripts utilizing custom algorithms or software that are central to the research but not yet described in published literature, software must be made available to editors and reviewers. We strongly encourage code deposition in a community repository (e.g. GitHub). See the Nature Portfolio guidelines for submitting code & software for further information.

Data

Policy information about availability of data

All manuscripts must include a data availability statement. This statement should provide the following information, where applicable:

- Accession codes, unique identifiers, or web links for publicly available datasets
- A description of any restrictions on data availability
- For clinical datasets or third party data, please ensure that the statement adheres to our policy

The datasets generated and analyzed during the current study are available from the corresponding author upon reasonable request.

Research involving human participants, their data, or biological material

Policy information about studies with human participants or human data. See also policy information about sex, gender (identity/presentation), and sexual orientation and race, ethnicity and racism.

Reporting on sex and gender	76.5% of participants were female (assigned sex at birth). Detailed demographic information are included in Supplemental Figure 1. We found no statistically significant effect of sex on the main study outcome (depression severity) or prediction accuracy and thus all analyses are aggregated across sexes. Sex was one of the factors impacting study adherence for participants receiving online care.
Reporting on race, ethnicity, or other socially relevant groupings	26.5% of participants self identified as white. Detailed demographic information are included in Supplemental Figure 1. We found no statistically significant effect of race/ethnicity on the main study outcome (depression severity) or prediction accuracy and thus all analyses are aggregated across race/ethnicity.
Population characteristics	Participants (N = 437; 76.5% female, 26.5% white) are University of California Los Angeles (UCLA) students experiencing mild to severe symptoms of depression or anxiety.
Recruitment	Recruitment and enrollment into the open trial occurred between Fall 2017 and Spring 2020. All University of California, Los Angeles (UCLA) students were made aware of the screener through announcements from UCLA's Chancellor, tabling at campus events, campus residence hall flyers, classroom announcements, web-based advertisements, banners, and email blasts.
Ethics oversight	The project was approved by the UCLA Institutional Review Board (approval numbers 17-001938, 16-001395, and 17-001365).

Note that full information on the approval of the study protocol must also be provided in the manuscript.

Field-specific reporting

Please select the one below that is the best fit for your research. If you are not sure, read the appropriate sections before making your selection.

☐ Life sciences ☒ Behavioural & social sciences ☐ Ecological, evolutionary & environmental sciences

For a reference copy of the document with all sections, see nature.com/documents/nr-reporting-summary-flat.pdf

Behavioural & social sciences study design

All studies must disclose on these points even when the disclosure is negative.

Study description	The data from this study come form an open trial. Quantitative longitudinal measurements of mood and behavior are collected for participants undergoing treatment for up to 40 weeks.
Research sample	This study involves an existing data set. Participants (N = 437; 76.5% female, 26.5% white) are University of California Los Angeles (UCLA) students experiencing mild to severe symptoms of depression or anxiety.
Sampling strategy	This study involves an existing data set. No sample size calculations were performed. All individuals with at least five mental health assessments in the original study were included.
Data collection	This study involves an existing data set. Digital behavioral phenotypes were collected using the AWARE framework.
Timing	STAND enrolled participants in two waves. The first wave enrolled participants from April 2017 to June 2018. The second wave of enrollment began at the start of the academic year in 2018 and continued for three years, during which time, from March 2020, a Safer-At-Home order was imposed in Los Angeles to control the spread of COVID-19.
Data exclusions	All individuals with less than five mental health assessments in the original study were excluded from this study in order to have enough data points for each individual to train a prediction model.
Non-participation	Nearly 5000 students were screened and 516 received care. Attrition for participants which received clinical care was linear over the follow-up period, with 1.7% of participants dropping out CAT-DI assessments within two weeks into the study. Attrition for participants that received online support was large two weeks into the study (33.5% of Wave 1 and 37.3% of Wave 2 participants) and linear for the remaining of the study.
Randomization	Participants were classified into treatment groups based on their depression and anxiety scores at baseline, which indicated the severity of symptoms in those domains. Participants that exhibited scores below the moderate depression range (CAT-DI score < 74) were offered internet-based cognitive behavioral therapy, which includes adjunctive support provided by trained peers or clinical psychology graduate students via video chat or in person. Participants that exhibited scores in the range of severe depression symptoms (CAT-DI score 75-100) or who endorsed current suicidality were offered in-person clinical care which included evidence-based psychological treatment with option for medication management.

Reporting for specific materials, systems and methods

We require information from authors about some types of materials, experimental systems and methods used in many studies. Here, indicate whether each material, system or method listed is relevant to your study. If you are not sure if a list item applies to your research, read the appropriate section before selecting a response.

Materials & experimental systems

n/a	Involved in the study
☒	☐ Antibodies
☒	☐ Eukaryotic cell lines
☒	☐ Palaeontology and archaeology
☒	☐ Animals and other organisms
☒	☐ Clinical data
☒	☐ Dual use research of concern
☒	☐ Plants

Methods

n/a	Involved in the study
☒	☐ ChIP-seq
☒	☐ Flow cytometry
☒	☐ MRI-based neuroimaging
